# Nrf2-and p53-inducible REDD2/DDiT4L/Rtp801L confers pancreatic β-cell dysfunction, leading to glucose intolerance in high-fat diet-fed mice

**DOI:** 10.1016/j.jbc.2025.110271

**Published:** 2025-05-21

**Authors:** Yukiho Yamada, Natsuho Urakawa, Hisato Tamiya, Shuya Sakamoto, Hiroki Takahashi, Naoki Harada, Tomoya Kitakaze, Takeshi Izawa, Shigenobu Matsumua, Eiji Yoshihara, Hiroshi Inui, Tomoji Mashimo, Ryoichi Yamaji

**Affiliations:** 1 Department of Applied Biological Chemistry, Graduate School of Agriculture, Osaka Metropolitan University, Sakai, Osaka, Japan; 2 Division of Applied Life Sciences, Graduate School of Life and Environmental Sciences, Osaka Prefecture University, Sakai, Osaka, Japan; 3 The Lundquist Institute for Biomedical Innovation at Harbor-UCLA Medical Center, Torrance, California, USA; 4 Graduate School of Veterinary Science, Osaka Metropolitan University, Izumisano, Osaka, Japan; 5 Department of Nutrition, Graduate School of Human Life and Ecology, Osaka Metropolitan University, Habikino, Osaka, Japan; 6 Department of Medicine, David Geffen School of Medicine at University of California Los Angeles, Los Angeles, California, USA; 7 Department of Health and Nutrition, Otemae University, Osaka, Japan; 8 Division of Animal Genetics, Laboratory Animal Research Center, Institute of Medical Science, University of Tokyo, Minato-ku, Tokyo, Japan; 9 Center for Research and Development of Bioresources, Osaka Metropolitan University, Sakai, Osaka, Japan

**Keywords:** pancreatic β-cell, DNA damage-inducible transcript 4-like (DDiT4L), gene knockout, nuclear factor erythroid 2-related factor 2 (Nrf2), oxidative stress, p53, streptozotocin (STZ), stress response, the regulated in development and DNA damage response 2 (REDD2), type 2 diabetes

## Abstract

Pancreatic β-cells play a critical role in glucose homeostasis by secreting insulin. Chronic oxidative stress causes β-cell dysfunction, including β-cell loss; however, the underlying mechanisms remain unclear. Here, we demonstrate the critical role of the regulated in development and DNA damage response 2 (REDD2/DDiT4L/Rtp801L) in β-cell dysfunction. In INS-1 β-cells, *Redd2* was induced by high glucose/palmitate or streptozotocin (STZ) exposure. Knockdown of *Redd2* attenuated STZ-induced loss of cell viability, while REDD2 overexpression reduced cell viability and p70S6K phosphorylation, suggesting the involvement of suppression of mTORC1 activation. STZ also activated the transcription factors nuclear factor erythroid 2-related factor 2 (Nrf2) and p53, and overexpression of these transcription factors synergistically induced *Redd2* expression. Reporter assays using the *Redd2* promoter (−2328/−1) and chromatin immunoprecipitation identified the functional binding sites for Nrf2 (EpRE2, −349/−340) and p53 (p53RE1, −90/−81) on the *Redd2* promoter. Purified recombinant p53 and Nrf2 bound directly. There were no noticeable changes in male global *Redd2*-knockout mice (C57BL/6J background), except for inguinal adipose tissue decrease when the mice were fed a standard diet. In contrast, when the mice were fed a high-fat diet (HFD), *Redd2*-knockout mice exhibited improved glucose tolerance relative to littermate controls. *Redd2*-knockout in HFD-fed mice increased β-cell mass due to reduced β-cell apoptosis and elevated plasma insulin concentrations, whereas insulin sensitivity remained unaffected. In both STZ-induced male and female and HFD-fed male models, β-cell-specific *Redd2*-knockout improved glucose tolerance without affecting insulin sensitivity. Our results identify REDD2 as a novel regulator of β-cell dysfunction under oxidative stress.

Pancreatic β-cells play a pivotal role in glucose homeostasis *via* insulin secretion. The quantity and quality (*i*.*e*., the ability to respond to glucose) of β-cells determine the amount of insulin secreted (1). In patients with type 2 diabetes mellitus (T2DM), pancreatic β-cell mass is reduced compared with that in healthy individuals (2). Although β-cell mass is regulated by both cell proliferation and cell death, an increase in apoptotic cell death is the predominant factor contributing to β-cell loss in patients with T2DM (3). Pancreatic β-cells are vulnerable to oxidative stress, as the expression levels of antioxidant enzymes, such as catalase, superoxide dismutase, and glutathione peroxidase, are considerably low (4, 5). Oxidative stress in β-cells is pathophysiologically induced by free fatty acid (6) and glucose (7), leading to β-cell dysfunction, including apoptosis (8). In experimental mouse models, a high-fat diet (HFD) is commonly used to induce physiological oxidative stress in β-cells (8). Streptozotocin (STZ), a pharmacological analog of glucose, is selectively imported into β-cells and causes oxidative stress and thus is also employed in β-cell studies (9, 10).

Under oxidative stress conditions, nuclear factor erythroid 2-related factor 2 (Nrf2) is released from the oxidative sensor Kelch-like ECH-associated protein 1 (Keap1), translocates to the nucleus, and acts as a transcription factor by binding to the electrophile response element (EpRE) (11). Nrf2 induces antioxidant enzymes and protects β-cells from oxidative stress-induced apoptosis (12, 13). Consumption of an HFD induces p53 activation in β-cells through chronic oxidative stress and subsequent persistent DNA damage (14). p53 is activated through phosphorylation and acetylation and induces cell growth arrest and apoptosis (15, 16).

The regulated in development and DNA damage response 2 (REDD2/DDiT4L/Rtp801L) is a stress response protein (17, 18, 19). REDD2 overexpression promotes oxidative stress and cell death in blood and neuronal cells (20, 21). Inhibition of mTORC1 signaling is involved in REDD2-induced neuronal cell death (21). REDD2 inhibits mTORC1 by associating with 14-3-3 proteins, which release Tuberous sclerosis proteins 1 and 2 (TSC1/2) and inactivate Ras homolog enriched in brain (Rheb), a small GTPase of mTORC1 (22, 23). Activation of mTORC1 signaling in β-cells improves glucose tolerance (24). Although the expression of *Redd2* is cooperatively induced by p53 and XBP1 in intestinal epithelial cells (25), and dominant-negative p53 suppresses the expression of *Redd2* in skin tumor cells (26), the mechanism underlying the induction of *Redd2* remains largely unclear. Furthermore, the physiological role of REDD2 has not been examined using gene knockout (KO) animals.

The mechanisms by which oxidative stress causes β-cell dysfunction have not yet been fully elucidated. A previous study using microarray analysis revealed an increase in *Redd2* in the pancreatic islets of mice treated with STZ (27). However, its role in β-cells under oxidative stress remains unclear. In the present study, we investigated the role of REDD2 in β-cells under oxidative stress conditions and the mechanism of its induction. Furthermore, we generated *Redd2* KO mice and examined their glucose tolerance under oxidative stress induced by HFD.

## Results

### REDD2 is increased under oxidative stress and decreases cell survival and insulin secretion in pancreatic β-cells

**Figure 1:**
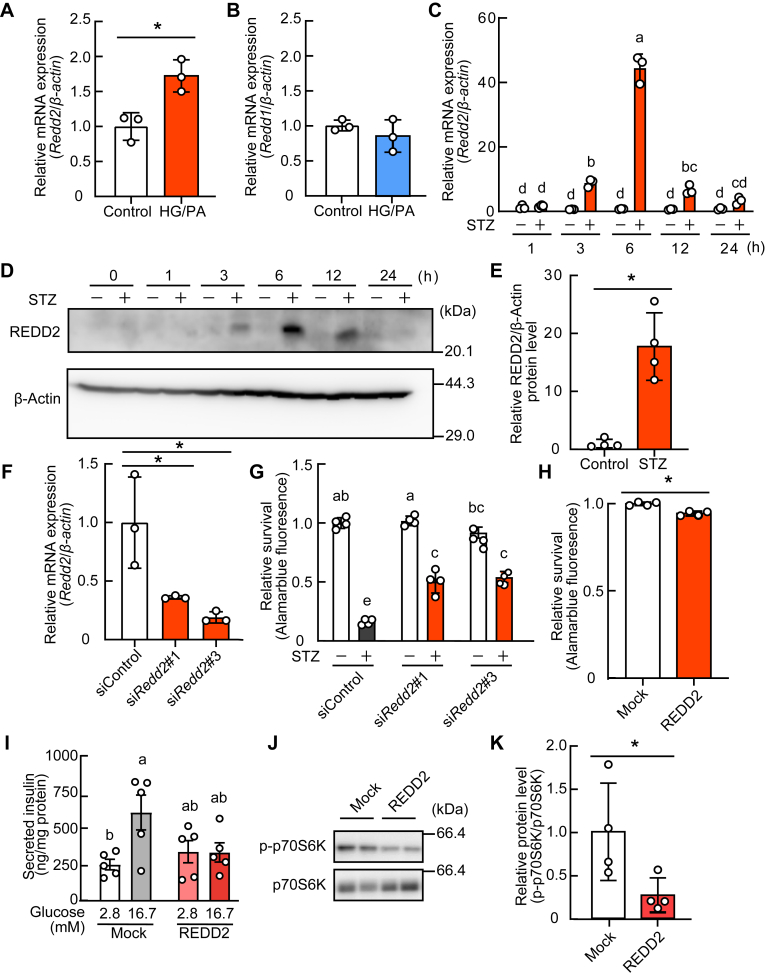
**Expression and function of the regulated development and DNA damage response 2 (REDD2) in INS-1 β-cells**. *A and B*, INS-1 cells were incubated with high glucose (22.2 mM) and palmitate (0.5 mM) for 6 h. *Redd2* (*A*) and *Redd1* (*B*) mRNA expression levels were determined by quantitative RT-PCR. *C–E*, INS-1 cells were incubated in the presence or absence of streptozotocin (STZ, 1 mM) for the indicated times. *Redd2* mRNA (*C*) and protein (*D*) levels were determined by quantitative RT-PCR and western blotting, respectively. Densitometric analysis of the Western blot data, using samples collected 6 h after stimulation (*E*). *F and G*, INS-1 cells were transfected with siRedd2#1 and siRedd2#3. *Redd2* expression, 6 h after STZ stimulation, was determined by quantitative RT-PCR (*F*). The cells were incubated with STZ (1 mM) for 24 h, followed by determination of cell viability using AlamarBlue dye (*G*). *H–J*, INS-1 cells were transfected with REDD2 expression vector. Cell viability was determined using AlamarBlue dye (*H*). Glucose-induced insulin secretion was quantified (*I*). Phosphorylation levels of p70S6K were determined by western blotting, followed by the calculation of band intensities (*J* and *K*). Data are expressed as mean ± SD (n = 4). *Asterisks* indicate statistically significant differences (*p* < 0.05, Student's *t* test or Dunnett’s test). *Different letters* indicate statistically significant differences (*p* < 0.05, Tukey-Kramer's test).

We examined the expression of REDD2 under oxidative stress using INS-1 β-cells. *Redd2*, but not *Redd1*, expression was increased by a high glucose/palmitate exposure (Fig. 1, *A* and *B*), which promotes oxidative stress in β-cells (28). STZ markedly increased *Redd2* mRNA expression in a time-dependent manner in INS-1 cells (Fig. 1*C*). A similar pattern of induction was also observed in REDD2 protein levels (Fig. 1*D*), with a significant increase after 6 h of stimulation (Fig. 1*E*). Subsequently, we examined whether increased REDD2 affected cell survival in INS-1 cells. Knockdown of *Redd2* by siRNA suppressed *Redd2* expression (Fig. 1*F*) and attenuated the STZ-induced loss of cell viability in INS-1 cells (Fig. 1*G*). Conversely, REDD2 overexpression caused a loss of cell viability (Fig. 1*H*) and decreased glucose-stimulated insulin secretion (Fig. 1*I*). Increased REDD2 also reduced the phosphorylation levels of p70S6K (Fig. 1, *J* and *K*), a substrate for mTORC1. These results indicate that the induction of REDD2 expression under oxidative stress attenuates cell survival and insulin secretion in INS-1 cells.

### Oxidative stress-induced Nrf2 and p53 induce Redd2 in β-cells

**Figure 2:**
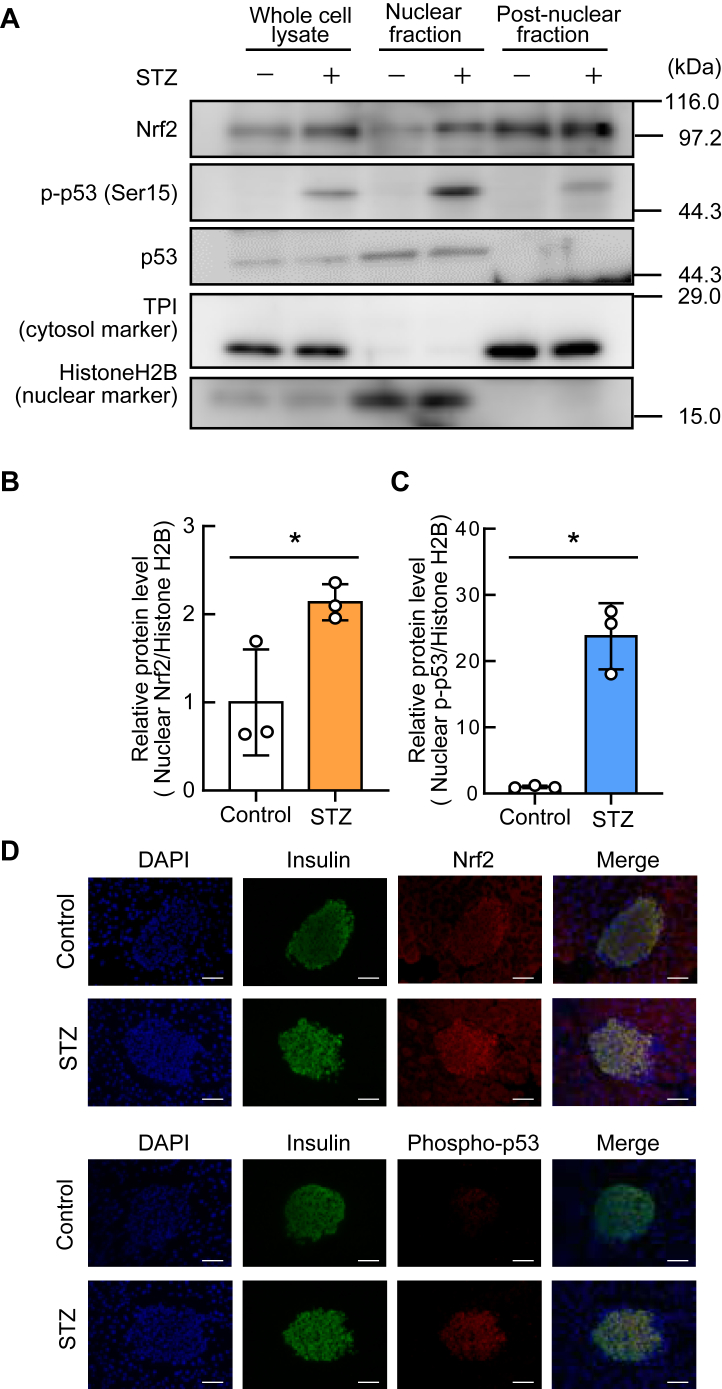
**Nuclear translocation of nuclear factor erythroid 2-related factor 2 (Nrf2) and p53 by oxidative stress in INS-1 cells and pancreatic β-cells in male mice**. *A*, INS-1 cells were incubated with or without 1 mM streptozotocin (STZ) for 3 h. After subcellular fractionation, the cell lysates were analyzed by western blotting using anti-Nrf2, anti-p-p53 (Ser15), anti-p53, anti-triosephosphate isomerase (TPI, cytosol marker), and anti-HistoneH2B (nuclear marker) antibodies. *B and C*, densitometric analysis of nuclear Nrf2 (*B*) and nuclear p-p53 (*C*). *D*, immunofluorescent analysis of pancreatic sections from mice treated with STZ (40 mg/kg, four consecutive days). The sections were stained for insulin (*green*), Nrf2 and p53 (*red*), and 4′,6-diamidino-2-phenylindole (DAPI, *blue*). Representative images from four independent experiments. Bar = 50 μm. Data are expressed as mean ± SD (n = 3). *Asterisks* indicate statistically significant differences (*p* < 0.05, Student’s *t* test).

Nrf2 and p53 are transcription factors involved in oxidative stress. We found that the nuclear localization of Nrf2 and phosphorylated p53 (p-p53) was increased by STZ-induced oxidative stress in INS-1 cells (Fig. 2, *A*–*C*). Similarly, increasing trend in the nuclear localization of Nrf2 and p-p53 were observed in β-cells in mice treated with STZ (Fig. 2*D*).

**Figure 3:**
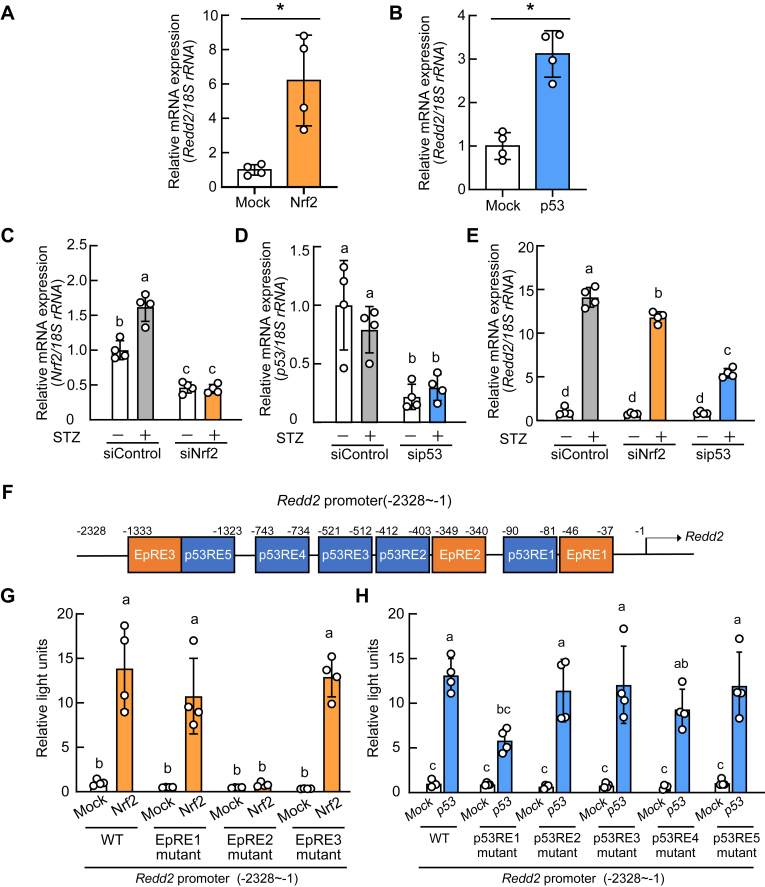
**Regulation of *the regulated development and DNA damage response 2* (*Redd2*) expression by nuclear factor erythroid 2-related factor 2 (Nrf2) and p53 in INS-1 β-cells.***A and B*, INS-1 cells transfected with Nrf2 (*A*) or p53 (*B*) expression vector were incubated for 12 h. *Redd2* mRNA expression was determined with quantitative RT-PCR. *C–E*, INS-1 cells transfected with siNrf2 or sip53 were incubated with or without of 1 mM streptozotocin (STZ) for 6 h. The mRNA expressions of *Nrf2* (*C*), *p53* (*D*), and *Redd2* (*E*) were determined using quantitative RT-PCR. *F*, Promoter analysis of putative electrophile response elements (EpREs) and putative p53 response elements (p53REs) in mouse *Redd2* promoter (−2328/−1) region. *G and H*, INS-1 cells transfected with Nrf2 or p53 expression vector, a firefly luciferase reporter vector containing the wild-type (WT) or mutant *Redd2* promoter (−2328/−1), and the *Renilla* luciferase reporter vector pGL4.73[*hRluc*/SV40] for 24 h. Luciferase activities were measured using the Dual-Luciferase Reporter Assay System. Data are expressed as mean ± SD (n = 4). Asterisks indicate statistically significant differences (*p* < 0.05, Student's *t* test). Different letters indicate statistically significant differences (*p* < 0.05, Tukey-Kramer's test).

Overexpression of either Nrf2 or p53 induced the expression of *Redd2* in INS-1 cells (Fig. 3, *A* and *B*). Knockdown of *Nrf2* and *p53* by siRNA not only decreased their expression levels (Fig. 3, *C* and *D*) but also decreased *Redd2* expression induced by STZ (Fig. 3*E*). In the *Redd2* promoter, three putative EpREs (EpRE1, EpRE2, and EpRE3) and five putative p53-response elements (p53RE: p53RE1, p53RE2, p53RE3, p53RE4, and p53RE5) were identified using LASAGNA-Search 2.0 (RRID:SCR_010883; Fig. 3*F*). After constructing vectors with mutations at these sites, we performed a reporter gene assay. As shown in Figure 3, *G* and *H*, the induction of reporter activity by Nrf2 and p53 was diminished by EpRE2 and p53RE1 mutations, respectively.

### Nrf2 and p53 cooperatively regulate Redd2 promoter activity

**Figure 4:**
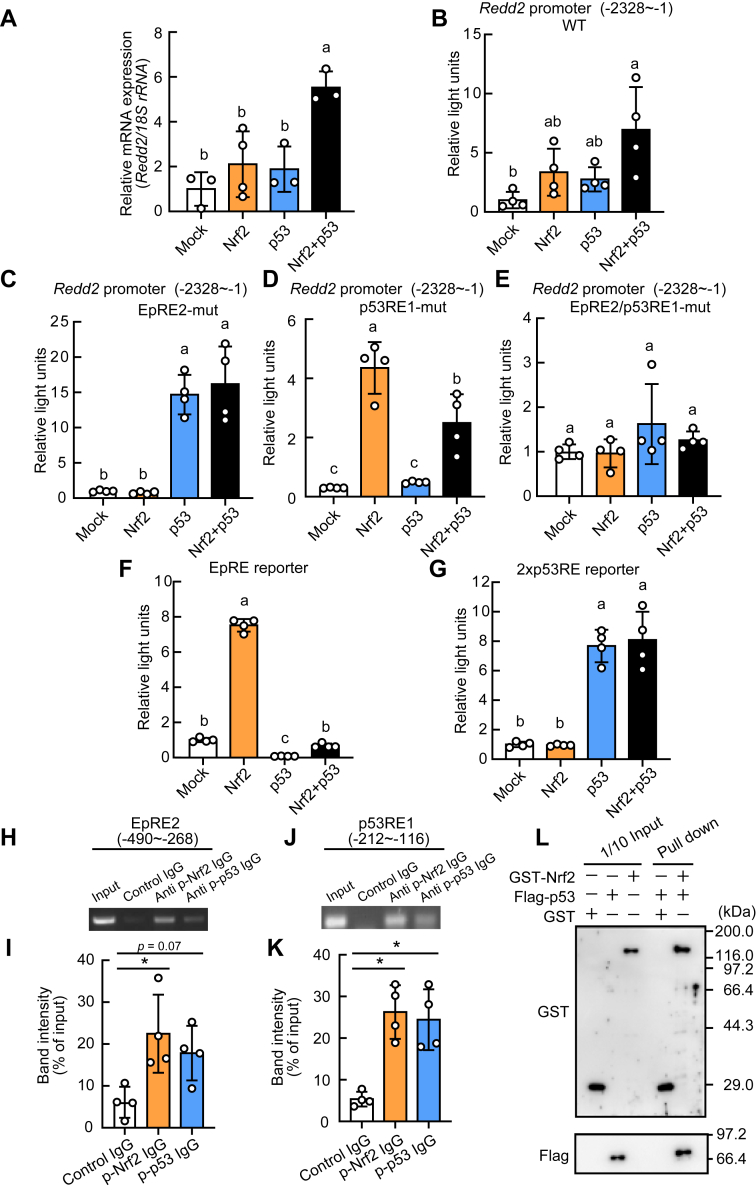
**Interaction of nuclear factor erythroid 2-related factor 2 (Nrf2) and p53 on the promoter of *the regulated development and DNA damage response 2* (*Redd2*) in INS-1 β-cells**. *A*, INS-1 cells were transfected with Nrf2 and/or p53 expression vector by electroporation for 24 h and *Redd2* expressions were determined. *B–G*, INS-1 cells were transfected with Nrf2 and/or p53 expression vector, a firefly luciferase reporter vector of wild type or mutant mouse *Redd2* promoter (−2328/−1) (WT (*B*), EpRE2 mutant (*C*), p53RE1 mutant (*D*), or EpRE2/p53RE1 mutant (*E*)), EpRE reporter vector (F), or 2xp53RE reporter (G) and the *Renilla* luciferase reporter vector (pGL4.74[*hRluc*/TK] or pGL4.73[*hRluc*/SV40]) for 24 h. Luciferase activities were determined using Dual-Luciferase reporter assay system. *H–K*, ChIP assay was performed using control IgG, anti-p-Nrf2 IgG, and anti-p-p53 IgG. The co-precipitated DNA was subjected to PCR using primers that amplify *Redd2* promoter containing the EpRE2 (*H*) or p53RE1 (*J*) elements in rat INS-1 cells. Densitometry of relative amplified DNA bands containing the EpRE2 (*I*) and p53RE1 (*K*). *L*, GST pull-down assay with purified GST or GST-Myc3-Nrf2 and His-Flag-p53. Pulled down samples were analyzed with western blotting with anti-GST and anti-Flag antibodies. Data are expressed as mean ± SD (n = 4). *Asterisk* indicates statistically significant differences (*p* < 0.05, Dunnett’s test). *Different letters* indicate statistically significant differences (*p* < 0.05, Tukey-Kramer's test).

We examined whether Nrf2 and p53 cooperate in regulating the expression of *Redd2*. Nrf2 and p53 synergistically increased *Redd2* mRNA levels (Fig. 4*A*) and *Redd2* promoter activity (Fig. 4*B*). In contrast, the synergistic effect on promoter activity was abolished with EpRE2- or p53RE1-mutant promoter (Fig. 4, *C* and *D*). The effects of Nrf2 and p53 were completely abolished in both EpRE2- and p53RE1-mutant promoters (Fig. 4*E*). Additionally, p53 suppressed Nrf2-activated EpRE reporter activity (Fig. 4*F*), while Nrf2 did not influence p53-induced p53RE reporter activity (Fig. 4*G*). In addition to anti-p-Nrf2 IgG, anti-p-p53 IgG also pulled down the *Redd2* promoter −490/−268 region containing EpRE2 (Fig. 4, *H* and *I*). Conversely, in addition to anti-p-p53 IgG, anti-p-Nrf2 IgG pulled down the *Redd2* promoter −212/−116 region containing p53RE1 (Fig. 4, *J* and *K*). The glutathione *S*-transferase (GST)-pull down assay with purified recombinant proteins showed that GST-Nrf2, but not GST, pulled down Flag-His-p53 (Fig. 4*L*). These results reveal a direct cooperative relationship between Nrf2 and p53 at the *Redd2* promoter.

### Redd2-KO in mice ameliorates HFD-induced glucose intolerance

**Figure 5:**
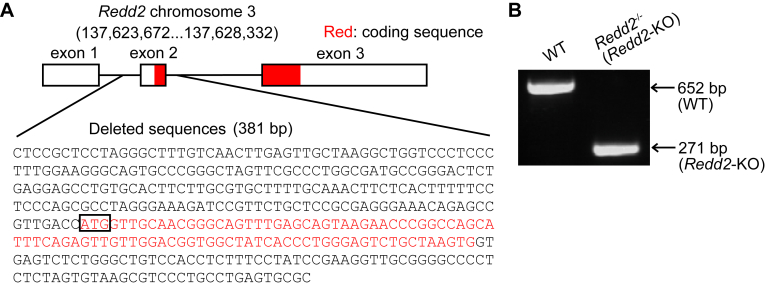
**Construction of *the regulated in dev*elopment and DNA damage response two knockout (Redd2-KO) mouse**. *A*, location of the deletion of chromosome three in *Redd2-KO* mice. *B*, genotyping results of wild-type and *Redd2*-KO mice.

To examine the role of REDD2 *in vivo*, we generated global *Redd2*-KO mice. The *Redd2* deletion locus, containing the start codon, is shown in Figure 5*A*. The deletion of the *Redd2* locus was confirmed by genotyping (Fig. 5*B*).

**Figure 6:**
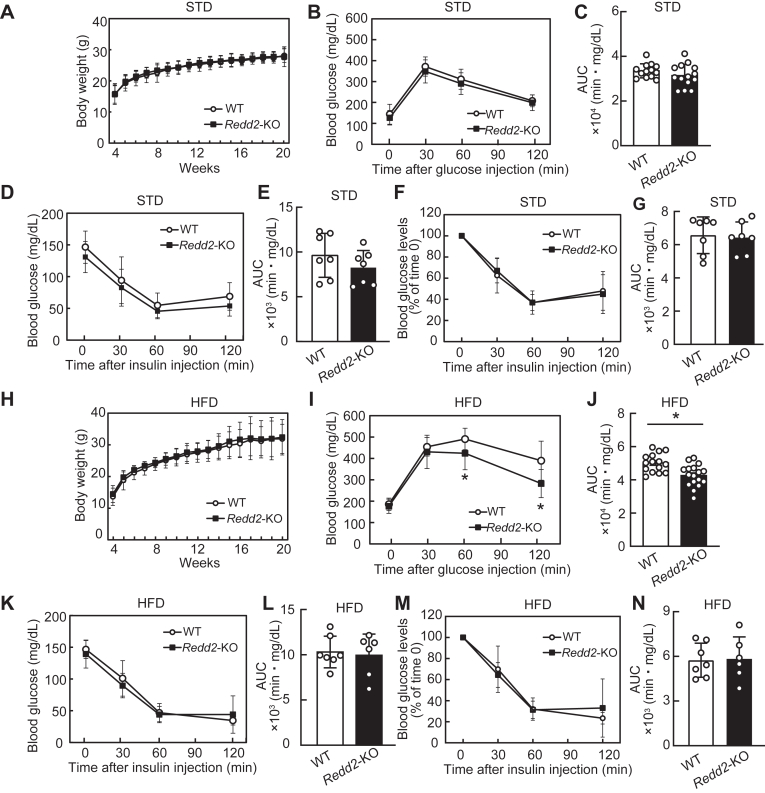
**Body weight and glucose and insulin tolerance in *the regulated in development and DNA damage response two* knockout (*Redd2*-KO) male mice**. *A and H*, body weight of mice fed standard diet (STD) (*A*, WT, n = 15; *Redd2*-KO, n = 18) or high-fat diet (HFD) (*H*, WT, n = 27; *Redd2*-KO, n = 19). *B*, *C*, *I*, *and J*, intraperitoneal glucose tolerance test in 16-week-old mice fed STD (*B*, WT, n = 13; *Redd2*-KO, n = 14) or HFD (*I*, WT, n = 15; *Redd2*-KO, n = 17) and their corresponding area under the curve (*C*, STD; *J*, HFD). *D–G and K–N*, insulin tolerance test in 17-week-old mice fed STD (*D* and *F* WT, n = 7; *Redd2*-KO, n = 7) or HFD (*K* and *M*, WT, n = 7; *Redd2*-KO, n = 6) and their corresponding area under the curve (*E* and *G*, STD; *L* and *N*, HFD). Raw data on blood glucose concentrations and blood glucose changes after insulin injection are shown. Data are expressed as mean ± SD and compared at the same time points. *Asterisks* indicate statistically significant differences (*p* < 0.05, Student’s *t* test).

*Redd2*-KO had no effect on body weight (Fig. 6*A*), glucose tolerance (Fig. 6, *B* and *C*), and insulin sensitivity (Fig. 6, *D*–*G*) when mice were fed a standard diet (STD). However, when mice were fed an HFD, *Redd2*-KO ameliorated glucose tolerance compared with that in the control (Fig. 6, *I* and *J*), despite showing no changes in body weight (Fig. 6*H*) or insulin sensitivity (Fig. 6, *K*–*N*). These results indicate that *Redd2*-KO alleviates HFD-induced glucose intolerance.

**Figure 7:**
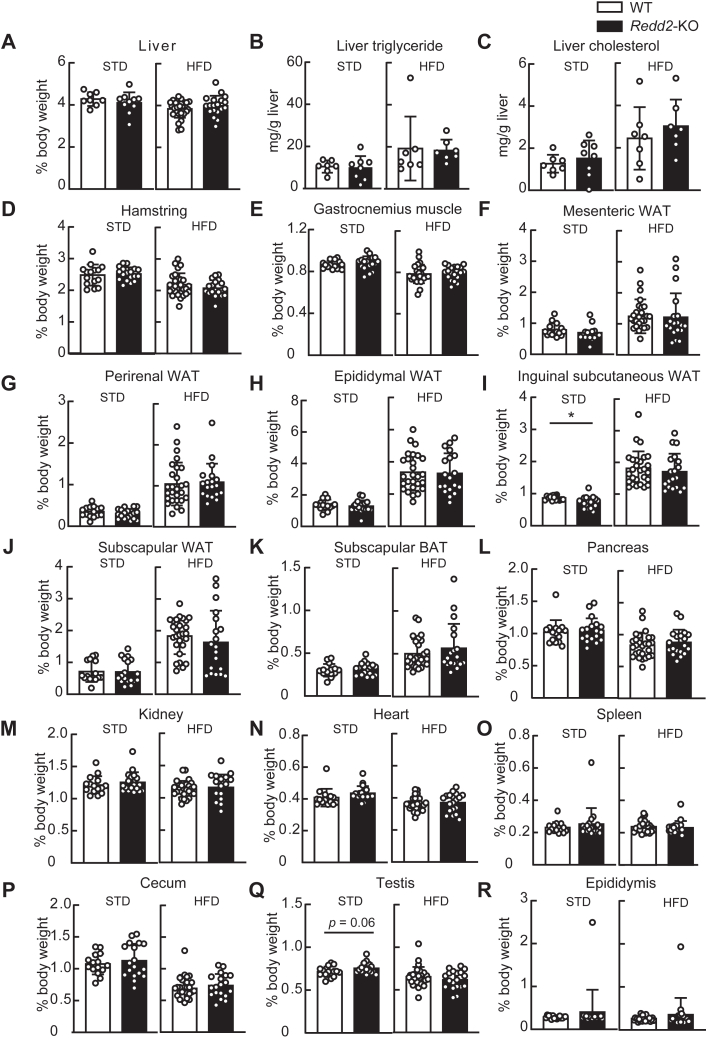
**Effect of *the regulated in development and DNA damage response two* knockout (*Redd2*-KO) on relative organ weight and liver lipid content in male mice**. *A*, *D–R*, weight of (*A*) liver, (*D*) hamstring, (*E*) gastrocnemius muscle, (*F*) mesenteric white adipose tissue (WAT), (*G*) perirenal WAT, (*H*) epididymal WAT, (*I*) inguinal subcutaneous WAT, (*J*) subscapular WAT, (*K*) subscapular brown adipose tissue (BAT), (*L*) pancreas, (*M*) kidney, (*N*) heart, (*O*) spleen, (*P*) cecum, (*Q*) testis, and (*R*) epididymis relative to body weight of mice fed standard diet (STD; WT, n = 15; *Redd2*-KO, n = 18) and fed high-fat diet (HFD; WT, n = 27; *Redd2*-KO, n = 19). *B and C*, liver triglyceride (*B*) and cholesterol (*C*) levels of mice fed STD (WT, n = 7; *Redd2*-KO, n = 8) and fed HFD (WT, n = 7; *Redd2*-KO, n = 7). Data are expressed as mean ± SD. *Different letters* indicate statistically significant differences (*p* < 0.05, Student’s *t* test).

The liver weight, triglyceride levels, and cholesterol levels in the liver were unaffected by *Redd2*-KO, regardless of diet (Fig. 7, *A*–*C*). Although *Redd2*-KO slightly decreased inguinal subcutaneous white adipose tissue (WAT) (Fig. 7*I*), it had no significant impact on the weight of muscles such as hamstring and gastrocnemius (Fig. 7, *D* and *E*); adipose tissues such as mesenteric WAT, perirenal WAT, epididymal WAT, subscapular WAT, and subscapular brown adipose tissue (Fig. 7*E*–*H*, 7*J*, and 7*K*); pancreas (Fig. 7*L*); kidney (Fig. 7*M*); heart (Fig. 7*N*); spleen (Fig. 7*O*); cecum (Fig. 7*P*); testis (Fig. 7*Q*); and epididymis (Fig. 7*R*) in both STD- and HFD-fed mice.

### Redd2-KO increases β-cell mass and its function in HFD-fed mice

**Figure 8:**
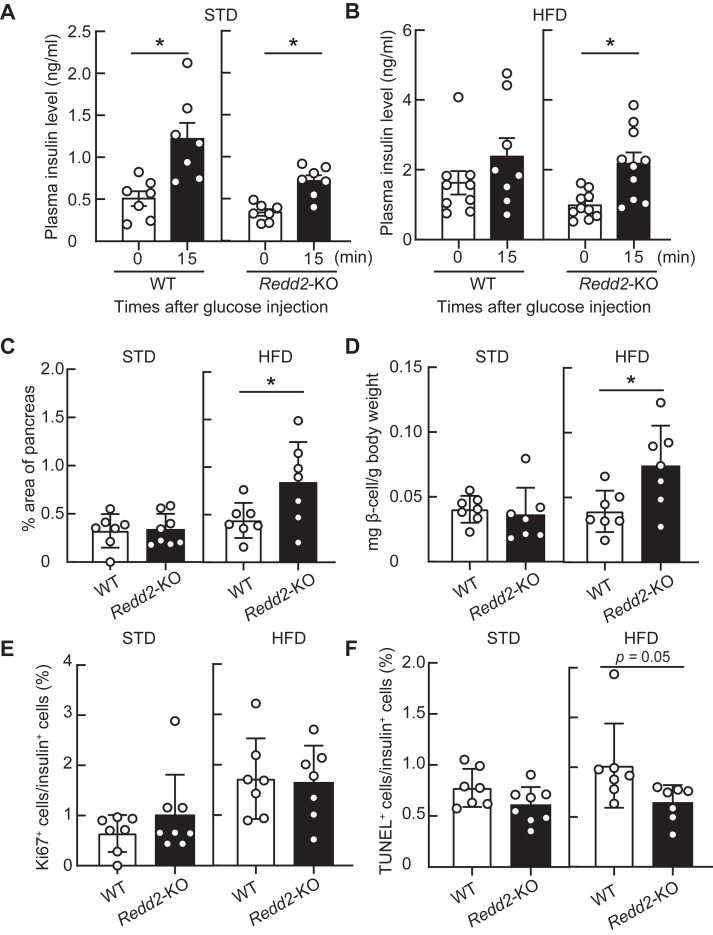
***Function and mass of pancreatic β-cells in the regulated development and DNA damage response two knockout (Redd2-KO) male mice.****A and B*, plasma insulin concentrations after glucose injection in mice fed a standard diet (STD; WT, n = 7; *Redd2*-KO, n = 7) or high-fat diet (HFD; WT, n = 9; *Redd2*-KO, n = 10). *C–F*, Insulin-positive cell area in total pancreas area (*C*), β-cell mass to body weight (*D*), β-cell proliferation (*E*), and β-cell apoptosis (*F*) in mice fed STD (WT, n = 7; *Redd2*-KO, n = 8), and fed HFD (WT, n = 7; *Redd2*-KO, n = 7). Data are expressed as mean ± SD. *Asterisks* indicate statistically significant differences (*p* < 0.05, Student’s *t* test).

In response to glucose injection, plasma insulin concentrations increased in both control and *Redd2*-KO mice fed STD (Fig. 8*A*). In contrast, when mice were fed an HFD, glucose-induced insulin increase was diminished in wild-type mice but maintained in *Redd2*-KO mice (Fig. 8*B*). The β-cell mass was evaluated by immunohistochemistry. Both the β-cell area in pancreatic sections and the ratio of β-cell mass to body weight were increased in *Redd2*-KO when mice were fed an HFD (Fig. 8, *C* and *D*). While *Redd2*-KO did not affect the proportion of Ki67-positive proliferative β-cells (Fig. 8*E*), it decreased the number of TUNEL-positive apoptotic β-cells (*p* = 0.05; Fig. 8*F*) in HFD-fed mice. Collectively, these results suggest that *Redd2*-KO increases β-cell mass by attenuating apoptosis, leading to improved glucose tolerance through enhanced insulin secretion.

### Pancreatic β-cell-specific Redd2-KO (βRedd2-KO) ameliorates glucose tolerance in STZ - or HFD-administered mice

**Figure 9:**
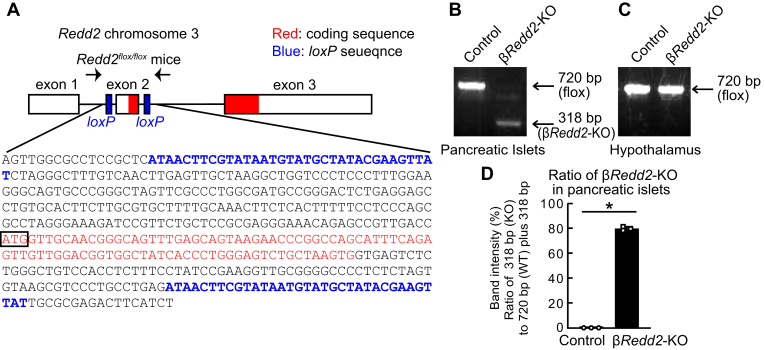
**Construction of pancreatic β-cell specific the regulated in development and DNA damage response two knockout (βRedd2-KO) mouse.***A*, location of *loxP* sequences in *Redd2*^*flox/flox*^ mice. *B and C*, genotyping results of the control (*Redd2*^*flox/flox*^) and β*Redd2*-KO (*Ins1*-*cre*; *Redd2*^*Δ/Δ*^) mice using genome DNA of pancreatic islets (*B*) or hypothalamus (*C*) of 8-week-old mice. *D*, estimation of knockout efficiency by calculating the intensity of PCR amplified bands from genomic DNA of pancreatic islets (n = 3).

We next investigated whether improved glucose tolerance could be observed in β-cell-specific deletion of *Redd2* (β*Redd2*-KO). To create β*Redd2*-KO mice, we generated *Redd2*^*flox/flox*^ *mice* and crossed them with *Ins1-cre* mice. The locations of the *loxP* sequences are shown in Figure 9*A*. β-cell-specific deletion of *Redd2* was addressed by genotyping using pancreatic islets (Fig. 9*B*), while no deletion was observed in non-target tissue such as the hypothalamus (Fig. 9*C*). Quantification of knockout efficiency revealed that approximately 80% of islet cells, consistent with the β-cell proportion within the islet, were knocked out (Fig. 9*D*), suggesting β-cell-specific and nearly complete *Redd2* deletion in β-cells.

**Figure 10:**
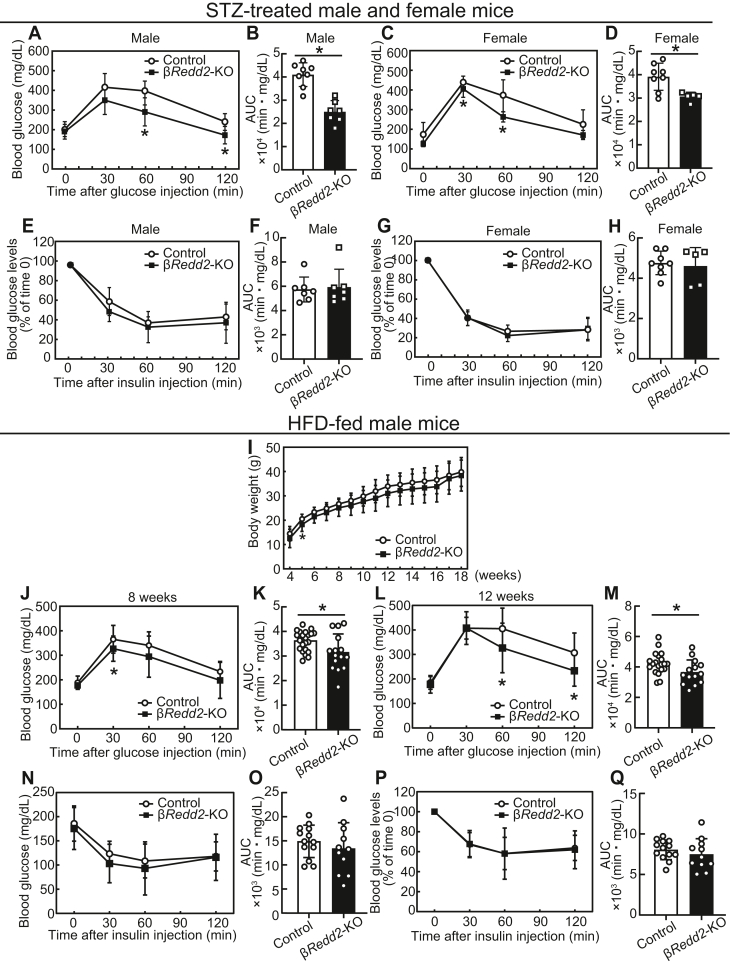
**Glucose and insulin tolerance in pancreatic β-cell specific the regulated in development and DNA damage response two knockout (βRedd2-KO) mice administrated with streptozotocin (STZ) or a high-fat diet (HFD).***A–H*, male (8 weeks of age) or female (7 weeks of age) control and β*Redd2*-KO mice were treated with STZ (40 mg/kg for four consecutive days). Intraperitoneal glucose tolerance test (*A*–*D*) and insulin tolerance test (*E*–*H*) (A and E, control male, n = 8; β*Redd2*-KO male, n = 7, *C* and *G*, control female, n = 8; β*Redd2*-KO female, n = 5) and their corresponding area under the curve (*B* and *F*, male; *D* and *H*, female). (*I*–*Q*) Male control and β*Redd2*-KO mice were fed with an HFD. *I*, Body weight (control, n = 22; β*Redd2*-KO, n = 16). *J–M*, intraperitoneal glucose tolerance test at 8 (*J*, control, n = 22; β*Redd2*-KO, n = 15) or 12 weeks of age (*L*, control, n = 20; β*Redd2*-KO, n = 14) and their corresponding area under the curve (*K* and *M*). Insulin tolerance test at 13 weeks of age. Raw data of blood glucose concentrations (*N*) and blood glucose changes after insulin injection (*P*) and their corresponding area under the curve (*O* and *Q*, respectively) are shown (control, n = 13; β*Redd2*-KO, n = 11). Data are expressed as mean ± SD and compared at the same time points. *Asterisks* indicate statistically significant differences (*p* < 0.05, Student’s *t* test).

In the STZ-induced diabetic model, β*Redd2*-KO mice exhibited improved glucose tolerance in both male (Fig. 10, *A* and *B*) and female (Fig. 10, *C* and *D*) mice, without changes in insulin tolerance in both sexes (Fig. 10, *E*–*H*). In an HFD-induced diabetic model in male mice, β*Redd2*-KO did not affect body weight (Fig. 10*I*) but significantly improved glucose tolerance at 8- and 12 weeks of age (Fig. 10, *J*–*M*). In contrast, β*Redd2*-KO did not affect insulin tolerance (Fig. 10, *N*–*Q*). These results highlight the importance of the REDD2 function in β-cells under stress conditions.

## Discussion

The present study shows that the expression of *Redd2* in β-cells was induced by Nrf2 and p53 under oxidative stress conditions and was negatively associated with cell viability through the inactivation of mTOR signaling. Global *Redd2*-KO ameliorated glucose intolerance by increasing β-cell mass and insulin concentrations without affecting insulin sensitivity in HFD-fed mice. In contrast, *Redd2*-KO had no notable effect on STD-fed young adult mice, except for a decrease in inguinal WAT, supporting the notion that *Redd2* functions as a stress response gene. To the best of our knowledge, this is the first study to investigate the role of REDD2 using gene KO mice. Our findings revealed that oxidative stress-induced REDD2 acts as a negative regulator of β-cell mass and regulation, contributing to glucose intolerance by decreasing secreted insulin. The results obtained from β*Redd2*-KO mice strongly support our findings. When we analyzed the data from human islets available at HumanIslets.com (29), *REDD2* expression was positively correlated with the culture period of islets and was negatively associated with glucose-stimulated insulin secretion and total islet equivalents after culture (Table S1). These findings indicate that our results also reflect the events in human β-cells.

REDD2 shares approximately 60% similarity (approximately 30% identity) with its homolog REDD1 in rodents and humans, although it lacks similarity to other proteins. The primary structure of REDD2 is highly conserved among rodents and humans, with >90% identity. REDD2 and REDD1 have common motifs and inhibit mTORC1 signaling (22, 30), although REDD1 possesses an additional inhibitory mechanism for mTORC1 signaling activated by G-protein coupled receptors (31). REDD1 is involved in stress responses across tissues as skeletal muscle, liver, retina, and kidney, and in the pathogenesis of diabetes (32). Although *Redd2* expression in skeletal muscle is relatively high compared to the expression in several other tissues (23), REDD2 cannot compensate for muscle atrophy in *Redd1*-KO mice (33). In this study, only *Redd2*, but not *Redd1*, was increased under oxidative stress from high-glucose/palmitate exposure. Moreover, in *Redd2*-KO mice, an improvement in β-cell function was observed, indicating that REDD1 cannot compensate for REDD2 in β-cells. Taken together, these findings indicate that REDD1 and REDD2 do not necessarily function in a compensatory manner. They are induced and function independently, depending on the tissue and environment.

The expression of *Redd2* was synergistically induced by Nrf2 and p53. HFD feeding activates both Nrf2 (34) and p53 (14) in the β-cells of mice. Similarly, STZ also activates Nrf2 in INS-1E cells (35) and p53 in β-cells of mice (27). Our results aligned with these observations. p53 affects the transcriptional activity of Nrf2 (36, 37), and Nrf2 increases the expression of p53 target gene (*i*.*e*., *p21*) (38). These results indicate that these two transcription factors affect each other. Although Nrf2 is generally thought to prevent β-cell death (12, 13), conflicting evidence suggests it may also contribute to apoptosis (39). In contrast, p53 is known to promote β-cell apoptosis (16, 40), though this role remains debated (41, 42). In hepatocarcinoma, p53 is required for an Nrf2-activator-induced apoptosis (36). Since apoptotic cell death was decreased in *Redd2*-KO mice, our results suggested that REDD2 mediated Nrf2 and/or p53-activated apoptosis.

In the *Redd2* promoter, EpRE2 (−349/−340) and p53RE1 (−90/−81) were responsible for induction by Nrf2 and p53, respectively, and are closely located. p53(R280K), but not wild-type p53, co-immunoprecipitates with Nrf2; however, the interaction surface of p53 (amino acids 98–128) does not contain the mutation site (43, 44). A recent study confirms that wild-type p53 and Nrf2 also co-immunoprecipitate from a crude cell lysate (45). We observed that wild-type p53 directly interacted with Nrf2. Thus, it is reasonable to assume that wild-type p53 has the ability to bind to Nrf2, though mutant p53(R280K) exhibits a stronger binding ability. Immunoprecipitation assays demonstrated that both Nrf2 and p53 bound to both EpRE2 (−349/−340) and p53RE1 (−90/−81). However, p53 does not regulate Nrf2 target genes *via* EpRE binding, nor does Nrf2 regulate the expression of p53 target genes through p53RE binding (37). Nrf2 and p53 did not exert a synergistic effect in either EpRE- or p53RE-mediated transcription. These findings suggest that EpRE2-bound Nrf2 and p53RE1-bound p53 can interact and cooperatively function at the *Redd2* promoter.

After induction in response to oxidative stress, REDD2 is rapidly degraded. The levels of REDD2 protein changed in parallel with its mRNA levels. Therefore, regulation of mRNA expression is critical for REDD2 expression. According to the analysis of AREsite2 (46), *Redd2* contains several putative AU-rich elements in its 3′ untranslated region. AU-rich element-mediated decay may be involved in the rapid decrease in *Redd2*.

HFD feeding induces metabolic stress such as glucotoxicity and lipotoxicity, leading to oxidative stress (47). In HFD-fed and *Db*/*Db* mice, metabolic stress elevates mTORC1 signaling in pancreatic β-cells (48). mTORC1 signaling in β-cells promotes β-cell survival and cell mass development (24, 48, 49, 50) and insulin secretion (49, 51), preventing β-cell dysfunction. In β-cells of global *Redd2*-KO mice fed with HFD, the apoptotic rate was decreased. Conversely, REDD2 overexpression suppresses the phosphorylation of p70S6K, a substrate of mTORC1, and increases the number of apoptotic cells. Therefore, the inhibition of mTORC1 likely contributes to REDD2-induced cell death and dysfunction in β-cells. This notion is supported by the observation that REDD2 overexpression promotes oxidative stress and cell death in the blood and neuronal cells (20, 21) by inhibiting mTORC1 (21). In the present study, we demonstrated that REDD2 negatively impacts β-cell health *in vitro* and *in vivo*. Therefore, our results provide evidence that REDD2 is a potential therapeutic target for treating T2DM.

## Experimental procedures

### Cell culture

INS-1 rat pancreatic β-cells (RRID:CVCL_0352) (52) were cultured in RPMI 1640 medium (Sigma-Aldrich, St Louis, MO, USA) supplemented with 10% fetal bovine serum (175012, Nichirei Biosciences, Tokyo, Japan), 10 mM HEPES, 1 mM sodium pyruvate, 100 U/ml sodium penicillin G, 100 μg/ml streptomycin sulfate, and 0.05 mM 2-mercaptoethanol. Cells were cultured in a humidified incubator set at 37°C with 5% CO_2_.

### Construction of plasmids

Plasmids expressing Myc-tagged Nrf2 (pcDNA3-Myc3-Nrf2) (53) and FLAG-tagged p53 (Flag-p53/pRK5) (54) were obtained from Addgene (Watertown, MA, USA). For constructing *Escherichia coli* expression vectors pET30-Flag-p53 and pGEX-Myc3-Nrf2, PCR products were amplified using the primers (Table S2) and either pcDNA3-Myc3-Nrf2 or Flag-p53/pRK5 as a template. The products were cloned into the *Bam*HⅠ and *Hind*Ⅲ sites of the pET30a (Novagen) or the *Eco*RⅠ and *Xho*Ⅰ sites of the pGEX5X-1 (Amersham Pharmacia Biotech) vectors using infusion cloning. To construct the mouse *Redd2* promoter (Gene ID: 73,284) reporter vector pGL-*Redd2*(−2328/−1)-Luc2, the *Redd2* promoter was amplified by nested PCR using specific primers (Table S2) and a mouse Genome Walker Kit *Sca*I Library (Takara Bio) as a template, followed by subcloning into the *Bgl*II site of the pGL4.14-TATA vector (55). *Redd2* promoter reporter vectors containing mutations in the EpRE and p53RE sites were constructed by site-directed PCR using specific primers (Table S2). The plasmids p3xFlag-REDD2 expressing FLAG-tagged rat REDD2 were constructed. *Redd2* (GenBank: XM_002729121) cDNA was amplified by nested PCR using specific primers (Table S2) and cDNA from INS-1 rat β-cells as a template. The PCR products were cloned into *Hind*III sites of p3xFLAG-Myc-CMV (Sigma-Aldrich) vector. p2xp53RE-TATA-Luc2 and pEpRE-TATA-Luc2 were constructed by inserting oligonucleotides (Table S2) containing two tandem repeats of p53RE1 from the mouse *Redd2* promoter (−110/−61) and the EpRE sequence from the human NAD(P)H quinone dehydrogenase promoter into the *Nhe*I and *Bgl*II sites of pGL4.14-TATA vectors (55).

### GST-pull down assay

pET30-Flag-p53 and pGEX-Myc3-Nrf2 were expressed in *E*. *coli* BL21 codon plus induced with 0.1 mM isopropyl β-d-thiogalactopyranoside at 30 °C for 5 h. His-FLAG-p53 was purified using phosphate buffer (20 mM KPB, pH 8.0, 500 mM NaCl, 20 mM imidazole). GST and GST-Myc3-Nrf2 were purified using RIPA buffer (50 mM Tris-HCl, pH 7.5, 500 mM NaCl, 2 mM EDTA, 1% TritonX-100, 1% sodium deoxycholate, 0.1% SDS, and 10% glycerol), followed by elution with either the phosphate buffer containing 500 mM imidazole or the RIPA buffer containing 50 mM reduced glutathione. After dialysis with PBS(−), the recombinant proteins were stored at −20 °C after adding the final 20% glycerol. Each recombinant protein (10 μg) was mixed and rotated overnight in pull-down buffer (20 mM HEPES-NaOH, pH 7.5, 150 mM NaCl, 0.5% Nonidet P-40, 2 mM EDTA, 10% glycerol, 1 mM phenylmethylsulfonyl fluoride, 10 μg/ml leupeptin, 1 μg/ml aprotinin, and 1 mM dithiothreitol). After binding to Glutathione Sepharose 4B (Cytiva), the resin was washed four times with pull-down buffer, followed by heating at 80 °C for 5 min. The supernatant was analyzed by western blotting.

### Luciferase reporter assay

INS-1 cells were seeded on 48-well plates at density of 4 × 10^4^ cells/well in culture medium without antibiotics. After incubation for 24 h, the cells were co-transfected with a firefly luciferase reporter vector, a *Renilla* luciferase reporter vector (pGL4.73[*hRluc*/SV40] or pGL4.74[*hRluc*/TK]; Promega), and either pcDNA3-Myc3-Nrf2 or Flag-p53/pRK5 using HilyMax (Dojindo) or Lipofectamine 3000 (Thermo Fisher Scientific) with Opti-MEM (Thermo Fisher Scientific) for 24 h. The amount of plasmid was kept constant by supplementing with the corresponding mock vector. The cells were harvested, and luciferase activities were determined using the Dual-Luciferase Reporter Assay System (Promega) and a GloMax 20/20 luminometer (Promega). Firefly luciferase activity values were normalized to that of *Renilla* luciferase activity (internal control) and expressed as relative light units.

### Subcellular fractionation

Subcellular fractionation was performed as previously described (56). Briefly, INS-1 cells were washed and harvested using a cellular fractionation buffer (20 mM HEPES-NaOH, pH 7.5, 250 mM sucrose, 1 mM EDTA, 1 mM sodium orthovanadate, and 10 mM sodium fluoride). The cells were resuspended in the cellular fractionation buffer supplemented with 10 μg/ml leupeptin, 1 μg/aprotinin, 1 mM phenylmethylsulfonyl fluoride, and 1 mM dithiothreitol and homogenized using a microhomogenizer with a power tool (PT-α; ISO). The homogenate (whole-cell lysate) was separated by centrifugation (1000×*g* for 10 min) to obtain nuclear pellets and post-nuclear fractions.

### Western blotting

Cells were lysed in lysis buffer (50 mM Tris-HCl, pH 7.5, 150 mM NaCl, 0.5% Nonidet P-40, 10 mM sodium pyrophosphate, 1 mM sodium orthovanadate, 10 mM sodium fluoride, 10 mM sodium molybdate, 2 mM EDTA, 1 mM phenylmethylsulfonyl fluoride, 10 μg/ml leupeptin, 1 μg/ml aprotinin, and 1 mM dithiothreitol). After sonication and centrifugation, the supernatant was subjected to SDS-PAGE. Western blotting was performed using rabbit monoclonal anti-p70S6 kinase (#2708, Cell Signaling Technology, RRID:AB_390722), rabbit monoclonal anti-phospho-p70S6K (#9234, Cell Signaling Technology, RRID:AB_2269803), rabbit polyclonal anti-Nrf2 (sc-13032, Santa Cruz Biotechnology, RRID:AB_2263168), rabbit polyclonal anti-p53 (bs-0033R, Bioss, Boston, MA, USA, RRID:AB_10855396), rabbit polyclonal anti-phospho-p53 (# 9284, Cell Signaling Technology, RRID:AB_331464), mouse monoclonal anti-DDIT4L (DDIT-03, Thermo Fisher Scientific, RRID:AB_2745588), rabbit polyclonal anti-triosephosphate isomerase (TPI) (57), rabbit polyclonal anti-Histone H2B (07-371, Millipore, RRID:AB_310561), or mouse monoclonal anti-β-actin (66009-1-Ig, Proteintech, RRID:AB_2687938) as the primary antibodies, and HRP-conjugated goat anti-rabbit or goat anti-mouse antibodies (170-6515, Bio-Rad, Hercules, CA, USA, RRID:AB_11125142 or 170-6516, Bio-Rad, RRID:AB_11125547) as secondary antibodies. Immunoreactive bands were developed using Immobilon Western Chemiluminescent HRP Substrate (Millipore) or Chemi-Lumi One Ultra (#11644, Nacalai Tesque). The bands were detected using an LAS4000 imager (Fujifilm). Band intensities were determined using Image J software version 1.53e.

### Overexpression and knockdown experiments

INS-1 cells were seeded on 48-well plates at a density of 8 × 10^4^ cells/well in medium without antibiotics and simultaneously reverse-transfected with siRNA using Lipofectamine RNAi MAX (Thermo Fisher Scientific) for 36 h. The siRNA sequences are as follows: siRedd2#1, sense: 5′-ACGUGAACUUGGAAAUUGAdTdT-3′ and antisense: 5′-UCAAUUUCCAAGUUCACGUdTdT-3′; siRedd2#3, 5′-CAUGCCAGAAUUUGGUUAAdTdT-3′ and antisense: 5′-AACCAAAUUCUGGCAUGdTdT-3′; siNrf2, sense: 5′-GGAUGAAGAGACCGGAGAAdTdT-3′ and antisense: 5′-UUCUCCGGUCUCUUCAUCCdTdT-3′; sip53, sense: 5′-GAAGAAAAUUUCCGCAAAAdTdT-3′ and antisense: 5′-UUUUGCGGAAAUUUUCUUCdTdT-3′. The control siRNA was obtained from Bioneer Corporation (SN-1013, Daejeon, Korea). Cells were treated with STZ (1 mM) for 6 h for gene expression analysis or 24 h for cell viability assays. INS-1 cells were transfected with pcDNA3-Myc3-Nrf2, Flag-p53/pRK5, p3xFlag-REDD2, or their corresponding mock vectors by electroporation (NEPA21 type II, Nepagene) for 12 or 24 h to determine *Redd2* expression or for 48 h to determine p70S6K activation. For determination of cell viability or secreted insulin, INS-1 cells were seeded on 96-well plates at 3 × 10^4^ cells/well in the antibiotic-free medium, reverse-transfected with p3xFlag-REDD2 or mock vector using Lipofectamine 3000 (Thermo Fisher Scientific) for 48 h. The medium was replaced with fresh medium containing 5% AlamarBlue (BUF012 B; Bio-Rad), and cells were incubated for 4 h. An excitation wavelength of 544 nm and emission wavelength of 590 nm were used to measure the fluorescence using a Fluoroscan Ascent FL instrument (Labsystems).

### Insulin secretion assay

The amount of insulin secreted by INS-1 cells was determined as previously described (58). Briefly, INS-1 cells were incubated with Krebs-Ringer bicarbonate buffer containing 0.1% bovine serum albumin and 2.8 mM or 16.7 mM glucose following transfection for 48 h. The amount of insulin secreted in the KRB buffer was measured using sandwich ELISA. Bovine insulin was used as the standard. The amount in insulin from INS-1 cells was normalized to the total protein concentration.

### Quantitative RT-PCR

Total RNA was extracted from cells using Sepasol-RNA I Super G reagent (09379–55, Nacalai Tesque), treated with DNase I (314-09071, Nippon Gene, Tokyo, Japan), and reverse-transcribed to cDNA using random hexamers, oligo(dT)20, and ReverTra Ace (TRT-101, TOYOBO). The resulting cDNAs were subjected to quantitative RT-PCR with TB Green *Premix Ex Taq* II DNA polymerase (RR820, Takara Bio) and specific primers (*Reddd2*, sense: 5′-GAAACAGAGCCGTTGACCAT-3′ and antisense: 5′-TTCAAACACCACCTCGTTGA-3′; *Redd1*, sense: 5′-CACCGGCTTCAGAGTCATCA-3′ and antisense: 5′-CGGGTCTCCACCACAGAAAT-3′; *β-actin*, sense: 5′-TGTCACCAACTGGGACGATA-3′ and antisense: 5′-GGGGTGTTGAAGGTCTCAAA-3′; *Nrf2*, sense: 5′-CACATCCAGACAGACACCAGT-3′ and antisense: 5′-CTACAAATGGGAATGTCTCTGC-3′; *p53*, sense: 5′-CATCTTCCGTCCCTTCTCAA-3′ and antisense: 5′-GTACCAGGTGGAGGTGTGGA-3′; *18S rRNA*, sense: 5′-GTAACCCGTTGAACCCCATT-3′ and antisense: 5′-GGCCTCACTAAACCATCCAA-3′) using a Thermal Cycler Dice TP850 (Takara Bio). The relative expression of each gene was calculated using the relative standard curve method and normalized to that of the control gene.

### Chromatin immunoprecipitation (ChIP) assay

The ChIP assay was performed as described previously (59, 60). Briefly, INS-1 cells were seeded on 100-mm dishes and treated with STZ (1 mM) for 3 h. The cells were fixed with 1% paraformaldehyde for 15 min at 37 °C, and the reaction was quenched by adding glycine at final concentration of 125 mM. Cells were collected and lysed in ChIP lysis buffer containing 10 μg/ml leupeptin, 1 μg/ml aprotinin, followed by sonication using Handy Sonic UR-21P (Tomy). The lysates were centrifuged, and the supernatants were diluted in ChIP dilution buffer. These were incubated overnight at 4 °C with 0.5 μg of rabbit monoclonal anti-phospho-Nrf2 (Ser40, EP1809Y, RRID:AB_1524049) or rabbit polyclonal anti-phospho-p53 (Ser15, Cat# 9284, Cell Signaling Technology, RRID:AB_331464) or control rabbit IgG (148-09551, FUJIFILM Wako, RRID_11125142). The mixture was further incubated with Dynabeads Protein G (Thermo Fisher Scientific) pre-blocked with bovine serum albumin and salmon sperm DNA (043–31381, FUJIFILM Wako) for 1 h. Proteins bound to the beads were sequentially washed with low salt wash buffer, high salt wash buffer, and LiCl wash buffer, followed by washing twice with TE buffer and elution with ChIP elution buffer. The eluted fraction or the sonicated lysate (50 μl, referred to as the input fraction) was incubated at 65 °C to dissolve cross-links, followed by digestion with RNase A and proteinase K. After phenol-extraction and ethanol precipitation, PCR was performed using 5 μl of the immunoprecipitated DNA from 50 μl DNA with the following specific primers: EpRE2, sense: 5′-GGACTGGAGCTGATGGGTTA-3′ and antisense: 5′-TCTTTGGTGTGAGCCCCTAC-3′; p53RE1, sense: 5′-CACCAACAACCAAACAGCAG-3′ and antisense: 5′-ACCTTTCAGTTCCTGCCAAG-3′.

### Animals

Mice were housed under controlled temperature conditions (23 ± 3 °C) with a 12:12-h light: dark cycle (lighting period starting at 08:00) and provided *ad libitum* access to STD (CE-2, CLEA Japan) and water. To generate *Redd2*^*flox*^ in mice (C57BL/6J background), *loxP* sequences were inserted flanking exon two of *Redd2* using the method described previously (61). *Redd2*-KO in mice deleting exon two of *Redd2* was obtained as by-product. β*Redd2*-KO mice were created using Cre-LoxP system employing *Ins1-cre* male mice (RBRC03934) (62), which was provided by the RIKEN BRC through the National BioResource Project of the MEXT/AMED. *Redd2*-KO were confirmed by PCR using the following primers: *Redd2*, sense: 5′-CCTTCAGCGTCTGGTGAAAT-3′ and antisense: 5′-AACTTCATCCCCAAGAGCCT-3′ and *cre*, sense: 5′-GTTTCACTGGTTATGCGGCGG-3′ and antisense: 5′-TTCCAGGGCGCGCGAGTTGATAG-3′; *Il2* (control for *cre*), sense: 5′-CTAGGCCACAGAATTGAAAGATCT-3′ and antisense: 5′- GTAGGTGGAAATTCTAGCATCATCC-3′. Genomic DNA isolated from the ear of global and β-cell specific *Redd2*-KO mice or isolated pancreatic islets or the hypothalamus of β*Redd2*-KO mice served as the template. The PCR product size of *Redd2* was 652 bp for wild-type, 271 bp for global *Redd2*-KO, 720 bp for *Redd2*^*flox*^, 318 bp for Cre-induced β*Redd2*^Δ^. For generating global *Redd2*-KO mice, heterozygous *Redd2*-KO male and female mice were mated, and the littermate wild-type and homozygous *Redd2*-KO male mice were used for experiments. For generating β*Redd2*-KO mice, *Ins1-cre*; *Redd2*^*flox/flox*^ male mice and *Redd2*^*flox/flox*^ female mice were mated, and *Ins1-cre*; *Redd2*^*flox/flox*^ (β*Redd2*-KO) and the littermate *Redd2*^*flox/flox*^ (control) were used for experiments (C57BL/6J background). Male 4-week-old mice were weaned and housed individually (cage dimensions: 136 × 208 × 115 mm; bedding material: clean chip, CLEA Japan). Wild type and global *Redd2*-KO mice were fed either STD (13% calories from fat) or an HFD (Quick Fat, 30% calories from fat and containing 25% sucrose, CLEA Japan) (63) until 20 weeks of age. β*Redd2*-KO were fed either STD (for STZ model) or an HFD (52.3% calories from fat and containing 10% sucrose) (64). Glucose and insulin tolerance tests were performed at 16 to 17 (for global *Redd2*-KO mice) or 8 to 13 (for HFD-fed β*Redd2*-KO mice) weeks of age. For the intraperitoneal glucose tolerance test (IPGTT) and insulin tolerance test (ITT), mice were fasted for 6 h (from 09:00) and then injected intraperitoneally with glucose at 2 g/kg body weight or insulin at 1 U/kg body weight. Blood glucose concentrations were determined from the tail vein using Stat Strip Glucose/Ketone meter (Nova Biomedical). To measure plasma insulin levels, mice were injected intraperitoneally with glucose (3 g/kg body weight). Blood was collected from the submandibular vein using hematocrit capillaries (Hirchemann-Laborgerate), and plasma was separated. Plasma insulin concentrations were determined using Ultra Sensitive Mouse Insulin ELISA Kit (Morinaga Institute of Biological Science Inc). After 4 h of fasting (from 09:00), mice were anesthetized by intraperitoneal injection of a mixed anesthetic agents solution containing medetomidine hydrochloride (0.75 mg/kg body weight, Nippon Zenyaku Kogyo Co., Ltd), midazolam (4.0 mg/kg body weight, Sandoz K.K., Tokyo, Japan), and butorphanol tartrate (5.0 mg/kg body weight, Meiji Animal Health Co., Ltd). The mice were euthanized by exsanguination from inferior vena cava, and blood and organs were harvested for further analysis. Wild-type and *Redd2*-KO mice were treated and dissected alternately. Male 7-week-old and female 6-week-old C57BL/6J mice were obtained from Kiwa Laboratory Animals (Wakayama, Japan) and acclimated for 1 week. STZ was diluted with 50 mM citrate buffer (pH 4.5) immediately prior to use. The mice were fasted for 4 h (from 10:00) and then injected intraperitoneally with STZ (40 mg/kg body weight) for four consecutive days. 10 and 12 days after the final treatment, IPGTT and ITT were performed, respectively. Five days after the final treatment, the mice were dissected after a 4 h fast (from 09:00) under anesthesia with the same mixed anesthetic solution. Perfusion fixation was performed using heparin sodium (10 U/ml PBS(−), FUJIFILM Wako) and 10% Formalin Neutral Buffer Solution (FUJIFILM Wako), and then the pancreas was collected. All animal experiments were approved by the Animal Care and Use Committee of Osaka Metropolitan University (formerly Osaka Prefecture University, Nos. 30–15, 19–44, 20–30, 21–23, 21–169, 22–14, 23–10, and 24–008). All procedures adhered to institutional guidelines and the ARRIVE guidelines.

### Immunohistochemistry and immunofluorescence microscopy

Pancreatic β-cell mass, cell proliferation, and cell death were determined as described previously (56). The pancreas was fixed with 10% formalin overnight, embedded in paraffin, and sliced into 4 μm sections. The sections were treated with H_2_O_2_ solution to inactivate endogenous peroxidases. Antigens were activated by heating in a 600 W microwave oven with 10 mM citrate buffer (pH 6.0), followed by incubation with PBS(−) containing 6% skim milk. For determining β-cell mass, the sections were immunostained with mouse monoclonal anti-insulin antibodies (D6C4cc, Hytest, RRID:AB_2858008) and Histofine Simple Stain Mouse MAX PO (Nichirei, RRID:AB_2819094). Immunoreactivity was visualized using a DAB peroxidase substrate kit (Vector Laboratories). To determine cell proliferation and apoptosis, sections were reacted with mouse monoclonal anti-insulin (D6C4cc) and rabbit monoclonal anti-Ki67 (SP6, Biocare Medical, RRID:AB_10949314) antibodies, followed by incubation with Goat anti-Mouse IgG (H + L) Cross-Adsorbed Secondary Antibody (Alexa Fluor 488, Thermo Fisher Scientific, RRID:AB_2534069) and Goat anti-Rabbit IgG (H + L) Cross-Adsorbed Secondary Antibody (Alexa Fluor 555, Thermo Fisher Scientific, RRID:AB_2535850) along with 4′,6-diamidino-2-phenylindole (DAPI). TUNEL staining was performed using DeadEnd Fluorometric TUNEL system kit (Promega). To analyze the expressions of p53 and Nrf2, the sliced pancreas sections from paraffin block were incubated with mouse monoclonal anti-insulin (D6C4cc) and rabbit polyclonal anti-Nrf2 (16396-1-AP, Proteintech, RRID:AB_2782956) or rabbit polyclonal anti-phospho-p53 (Ser15) (#9284, Cell Signaling Technology, RRID:AB_331464), followed by incubation with goat anti-mouse IgG (H + L) Cross-Adsorbed Secondary Antibody (Alexa Fluor 488) and goat anti-rabbit IgG (H + L) Cross-Adsorbed Secondary Antibody (Alexa Fluor 555) along with DAPI. The tissue sections were observed using a model BZ-X810 HS All-in-one Fluorescence Microscope (Keyence). The areas of the pancreas and insulin-positive cells were calculated using Image-Pro Premier image analysis software (Media Cybernetics, RRID:SCR_016497). β-cell area was calculated from the area percentage of insulin-positive cells in the pancreas. β-Cell mass was calculated as β-cell weight per mouse body weight. Cell proliferation or apoptosis were determined by measuring the ratio of both insulin and Ki67-positive cells or both insulin and TUNEL-positive cells to insulin-positive cells in the pancreas sections using Image J version 1.53e (National Institutes of Health, RRID:SCR_003070).

### Statistical analyses

Data were analyzed using Student’s *t* test (unpaired, two-tailed) or one-way analysis of variance with Tukey-Kramer’s or Dunnett’s *post hoc* tests using JMP statistical software version 8.0.1 (SAS Institute). For the *in vitro* study, biological replicates with experimental reproducibility are shown. Data are displayed as mean ± standard deviation (SD). *p <* 0.05 was defined as statistically significant.

## Data availability

Data are available within the article or the supplementary materials.

## Supporting information

This article contains supporting information.

## Conflict of interest

The authors declare that they have no conflicts of interest with the contents of this article.
