# Supplementary material for: Nrf2-and p53-inducible REDD2/DDiT4L/Rtp801L confers pancreatic β-cell dysfunction, leading to glucose intolerance in high-fat diet-fed mice
Source: J Biol Chem. 2025 May 21;301(6):110271. doi: 10.1016/j.jbc.2025.110271 (PMC12206130; doi:10.1016/j.jbc.2025.110271)
Supplement: Table S2 [file mmc2.docx]

Table S2. Primers and oligonucleotides for construction of vectors

| Vector |  | Sequences of primers or oligonucleotides |
| --- | --- | --- |
| pET30-Flag-p53 | Fw  Rv | 5′-GCTGATATCGGATCCGACTACAAGGACGACGATGAC-3′  5′-GTGCGGCCGCAAGCTTTCAGTCGACGTCTGAGTCAG-3′ |
| pGEX-Nrf2 | Fw  Rv | 5′-GTGGGATCCCCGAATTCGAGCAAAAGCTCATCTCAGAG-3′  5′-GATGCGGCCGCTCGAGCTATAGAATAGGGCCCTCTAG-3′ |
| pGL-*Redd2*  (-2328/-1)-Luc2  (1^st^ PCR) | Fw  Rv | 5′-TTGCAGACACATCAGTTACATTGC-3′  5′-CTTCGGATAGGAAAGAGGTGGACAG-3′ |
| pGL-*Redd2*  (-2328/-1)-Luc2  (2^nd^ PCR) | Fw  Rv | 5′-GAGATCTGTGAAGTGTGACAGGCAAGC-3′  5′-GAGATCTGCGCCAACTAAAGCCAACAG-3′ |
| pGL-*Redd2*  (-2328/-1)-Luc2-EpRE1-mut | Fw  Rv | 5′-GTGACCTGACCCTATAGGGGCGGGCCG-3′  5′-CGGCCCGCCCCTATAGGGTCAGGTCAC-3′ |
| pGL-*Redd2*  (-2328/-1)-Luc2-EpRE2-mut | Fw  Rv | 5′-CAGAATGACACAATAAAGTTTTGGGTC-3′  5′-GACCCAAAACTTTATTGTGTCATTCTG-3′ |
| pGL-*Redd2*  (-2328/-1)-Luc2-EpRE3-mut | Fw  Rv | 5′-GTGCCTGAGCCTATCTGACCTTGAGCA-3′  5′-TGCTCAAGGTCAGATAGGCTCAGGCAC-3′ |
| pGL-*Redd2*  (-2328/-1)-Luc2-p53RE1-mut | Fw  Rv | 5′-CAACTCCCAGGGGATTACCTGGGCCCCTG-3′  5′-CAGGGGCCCAGGTAATCCCCTGGGAGTTG-3′ |
| pGL-*Redd2*  (-2328/-1)-Luc2-p53RE2-mut | Fw  Rv | 5′-GGGTTAGAGTCAGAAAATAGGCATAAAGGATGC-3′  5′-GCATCCTTTATGCCTATTTTCTGACTCTAACCC-3′ |
| pGL-*Redd2*  (-2328/-1)-Luc2-p53RE3-mut | Fw  Rv | 5′-GTAGTATCCCCAAAAATACCCCATTGATTCC-3′  5′-GGAATCAATGGGGTATTTTTGGGGATACTAC-3′ |
| pGL-*Redd2*  (-2328/-1)-Luc2-p53RE4-mut | Fw  Rv | 5′-CTTCTTCCCAAAAAAACCCACCGGGAAC-3′  5′-GTTCCCGGTGGGTTTTTTTGGGAAGAAG-3′ |
| pGL-*Redd2*  (-2328/-1)-Luc2-p53RE5-mut | Fw  Rv | 5′-TCCCGTGCCTGAGACTACCTGACCTTGAG-3′  5′-CTCAAGGTCAGGTAGTCTCAGGCACGGGA-3′ |
| p3xFlag-REDD2  (1^st^ PCR) | Fw  Rv | 5′-GTCCAAGCATCGGGCTAATAG-3′  5′-GGTTTATTAGCCACTCATTAGGGAC-3′ |
| p3xFlag-REDD2  (2^nd^ PCR) | Fw  Rv | 5′-GAAGCTTATGGTTGCAACGGGCAGTTTG-3′  5′-GAAGCTTTCAGCACTCCTCAATGACTGTC-3′ |
| p2xp53RE-TATA-Luc2 | Fw  Rv | 5′-CCTGAGCTCGCTAGCGGCTGATCACCAACTCCCAGGGGCTTGCCT  GGGCCCCTGCCTGGGAAGGAGGCTGATCACCAACTCCCAGGGGCTTGCCTGGGCCCCTGCCTGGGAAGGA-3′  5′-GCCGAGGCCAGATCTTCCTTCCCAGGCAGGGGCCCAGGCAAGCC  CCTGGGAGTTGGTGATCAGCCTCCTTCCCAGGCAGGGGCCCAGGCAAGCCCCTGGGAGTTGGTGATCAGCC-3′ |
| pEpRE-TATA-Luc2 | Fw  Rv | 5′ -CTAGCGCAGTCACAGTGACTCAGCAGAATCA-3′  5′ -GATCTGATTCTGCTGAGTCACTGTGACTGCG-3′ |
